# SARS-CoV-2 and the ocular surface: test accuracy and viral load

**DOI:** 10.5935/0004-2749.2022-0172

**Published:** 2023-03-08

**Authors:** Dalton de Freitas Santoro, Flavio Eduardo Hirai, Lucas Baldissera Tochetto, Danielle Dias Conte, Ana Luísa Hofling Lima, Luciene Barbosa de Sousa, Nancy Cristina Junqueira Bellei, Denise Freitas, Lauro Augusto de Oliveira

**Affiliations:** 1 Department of Ophthalmology and Visual Science, Escola Paulista de Medicina, Hospital São Paulo, Universidade Federal de São Paulo, São Paulo, SP, Brazil; 2 Department of Medicine, Discipline of Infectious and Parasitic Diseases, Escola Paulista de Medicina, Hospital São Paulo, Universidade Federal de São Paulo, São Paulo, SP, Brazil

**Keywords:** COVID-19, SARS-CoV-2, Conjunctiva, Tears, Reverse transcriptase polymerase chain reaction, RNA, viral, COVID19, SARS-CoV-2, Túnica conjuntiva, Lágrimas, Reaçao em cadeia da polimerae via transcriptase reversa, RNA, viral

## Abstract

**Purpose:**

This study aimed to evaluate the pre-sence of severe acute respiratory syndrome coronavirus 2 (SARS-CoV-2) RNA in the ocular surface of individuals clinically suspected of coronavirus disease 2019 (COVID-19) and determine the accuracy of different approaches of molecular testing on the ocular surface based on the nasopharyngeal positivity status for COVID-19.

**Methods:**

A total of 152 individuals with suspected COVID-19 symptoms who simultaneously underwent nasopharyngeal and two different tear film collection techniques for quantitative reverse-transcriptase polymerase chain reaction (RT-qPCR) were included. Tears were collected and randomized: one eye had the filter strip for the Schirmer test and the contralateral eye had conjunctival swab/cytology in the inferior fornix. All patients underwent slit lamp biomicroscopy. The accuracy of various ocular surface collection techniques used for the detection of SARS-CoV-2 RNA was determined.

**Results:**

Of the 152 patients enrolled in the study, 86 (56.6%) had COVID-19 confirmed by nasopharyngeal PCR. Both tear film collection techniques detected viral particles: the Schirmer test was positive in 16.3% (14/86) and the conjunctival swab/cytology in 17.4% (15/86), with no statistically significant differences. No positive ocular tests were found among those with negative nasopharyngeal PCR tests. The overall agreement of the ocular tests was 92.7%, and in combination, the sensitivity would increase to 23.2%. The mean cycle threshold values in the nasopharyngeal, Schirmer, and conjunctival swab/cytology tests were 18.2 ± 5.3, 35.6 ± 1.4, and 36.4 ± 3.9, respectively. Compared with the nasopharyngeal test, the Schirmer (p=0.001) and conjunctival swab/cytology (p<0.001) tests had significantly different Ct values.

**Conclusion:**

The Schirmer (16.3%) and conjunctival swab (17.4%) tests were comparably capable of detecting SARS-CoV-2 RNA in the ocular surface by RT-PCR accurately based on nasopharyngeal status and demonstrated indistinct sensitivity and specificity. Simultaneous specimen sampling and processing from the nasopharyngeal, Schirmer, and conjunctival swab/cytology tests demonstrated significantly lower viral load in both ocular surface approaches than in the nasopharyngeal test. Ocular manifestations detected by slit lamp biomicroscopy were not associated with ocular RT-PCR positivity.

## Abstract (pt)

**Objetivo:**

Avaliar a presença de RNA de coronavírus 2 causador de síndrome respiratória aguda grave (SARS-CoV-2) na superfície ocular de indivíduos clinicamente suspeitos com COVID-19 e determinar a precisão de diferentes abordagens de testes moleculares na superfície ocular com base no status de positividade do RT-qPCR de nasofaringe para COVID-19.

**Métodos:**

152 indivíduos com sintomas suspeitos para a COVID-19 foram submetidos a coleta de reação em cadeia da polimerase de nasofaringe simultaneamente a duas técnicas diferentes de coleta de filme lacrimal para RT-qPCR: aleatoriamente, um olho com a tira filtro do teste de Schirmer e, o olho contralateral, com citologia (swab) conjuntival no fórnice inferior. Todos os indivíduos foram submetidos à biomicroscopia com lâmpada de fenda.

**Resultados:**

Dos 152 pacientes, 86 (56,6%) tiveram a COVID-19 confirmada por PCR de nasofaringe. Ambas as técnicas de coleta detectaram partículas virais: o teste de Schirmer foi positivo em 16,3% (14/86) e a citologia conjuntival em 17,4% (15/86), sem diferenças estatisticamente significativas. Não houve testes oculares positivos entre aqueles com reação em cadeia da polimerase de nasofaringe negativo. A concordância geral dos testes oculares foi de 92,7% e, em combinação, a sensibilidade aumentaria para 23,2%. Os valores médios do *limiar de ciclo* nos testes de nasofaringe, Schirmer e citologia conjuntival foram 18,2 ± 5,3, 35,6 ± 1,4 e 36,4 ± 3,9, respectivamente.

**Conclusão:**

Os testes de Schirmer (16,3%) e swab conjuntival (17,4%) foram igualmente capazes de detectar RNA de SARS-CoV-2 na superfície ocular por RT-PCR e demonstraram sensibilidade e especificidade indistintas. A coleta simultânea de amostras ao processamento dos testes de RT-PCR de nasofaringe, Schirmer e citologia (swab) conjuntival demonstraram carga viral significativamente menor em ambas as abordagens da superfície ocular em comparação com o teste de nasofaringe. As manifestações oculares detectadas pela biomicroscopia com lâmpada de fenda não foram claramente associadas à positividade do RT-PCR ocular.

## INTRODUCTION

Severe acute respiratory syndrome coronavirus 2 (SARS-CoV-2) belongs to the coronavirus family and was identified as the causative agent of coronavirus disease 2019 (COVID-19)^(1)^. The coronavirus family is known for transmission through contact and inhalation of droplets and aerosols expelled by patients with infection. Despite using the same human angiotensin-converting enzyme 2 (ACE2) receptor to cell invasion, the transmission rate of SARS-CoV-2 is higher than that of other viruses in the same family, such as the ones causing the outbreaks of SARS in 2003^(2)^ and Middle East respiratory syndrome in 2012^(3)^.

This increased transmission rate may be caused by the presence of other receptors that allow greater penetration of SARS-CoV2, such as the transmembrane serine protease 2 (TMPRSS2) and transmembrane glycoprotein CD147, which have already been detected on the ocular surface^(4-7)^. Although the ocular surface meets the pathophysiological conditions necessary for SARS-CoV-2 invasion, the level of evidence of conjunctival transmission and viral shedding through tears is insufficient and controversial. To date, several studies regarding SARS-CoV-2 viral particles on the ocular surface with variable positivity (0%-27.7%) have been published. In a literature review, Emparan et al. reported a variable PCR positivity (0%-7.14%) in both the tear (Schirmer test) and conjunctiva (conjunctival swab)^(7)^. Dutescu et al. found viral RNA in the tear film of 5 of 18 (27.7%) hospitalized patients^(8)^. The accuracy of different techniques for the investigation of SARS-CoV-2 on the ocular surface (Schirmer test, tear film, and conjunctival swab) is not clearly estimated and compared^(9)^.

The cycle threshold (Ct) is a semiquantitative value that can be used as an approximate proxy for the viral load. Ct can be defined as the thermal cycle number in a typical reverse-transcriptase polymerase chain reaction (RT-PCR) assay with a maximum of 40 thermal cycles. The lower the Ct value, the higher the quantity of the viral genetic materials in the sample.

This study aimed to investigate the presence of SARS-CoV-2 RNA in the tears and on the ocular surface of individuals with clinically suspected COVID-19 and determine the accuracy of different approaches of sample collection on the ocular surface based on nasopharyngeal COVID-19 positivity status.

## METHODS

From June to July 2020, 152 individuals with suspected COVID-19 symptoms were examined in an outpatient clinic. The study protocol was approved by the Ethics Committee of Universidade Federal de São Paulo (CEP: 0442/2020), and all patients provided written informed consent before participation. The study followed the ethical principles of the Declaration of Helsinki.

A face-to-face questionnaire was used to identify the date of disease onset and evaluate the presence of general signs and symptoms, such as fever, cough, difficulty breathing, body pain, headache, smell changes (anosmia), and taste changes (dysgeusia), and to determine ocular symptoms, such as redness, tearing, photophobia, eye discharge, itching, foreign body sensation, altered visual acuity, and eyelid edema. All patients underwent slit lamp biomicroscopy,

All patients were tested for SARS-CoV-2 by nasopharyngeal molecular test to confirm or exclude COVID-19.

### Collection and processing of ocular surface specimens

Ocular samples were obtained randomly between eyes, according to a sequence in a randomization table created by an appropriate statistical software (Stata Corp V.14, College Station, TX, USA) and simultaneously from the nasopharynx, with one kit for each (kit specifications are mentioned below). In one eye, the tear film was collected with the filter strip of the Schirmer test (Ophthalmos Rohto, São Paulo, Brazil) placed in the inferior conjunctival fornix, without anesthetic eye drops, for 3 min or time enough to moisten the filter strip until achieving 15 mm and, in the contralateral eye, with conjunctival swab/cytology in the inferior fornix performed under topical anesthesia with a cervical brush with soft nylon bristles (KOL16999A, Kolplast Inc., São Paulo, Brazil). Protective concerns between patients’ sampling were conducted, including changing gloves and using 70% ethyl alcohol to avoid cross-contamination. Samples from each eye were immediately stored in 150 µL of storage and stabilization solution (DNA/RNA Shield-Zymo Commercial Kit). All samples were adequately stored at -80°C. Processing and analysis of samples were performed at the Clinical Virology Laboratory-Federal University of São Paulo after sample collection; therefore, the storage time varied between samples. They were subsequently used for RNA purification using the Quick-RNA™ Viral Kit (Zymo Research, Irvine, CA, USA). Molecular detection of SARS-CoV-2 was performed by quantitative reverse-transcriptase polymerase chain reaction (RT-qPCR) using the XGEN MASTER COVID-19™ Kit (Mobius Life Science, Paraná, Brazil), with the detection of the *ORF1ab* and *N* genes for SARS-CoV-2. All inconclusive samples were reanalyzed using the GeneFinder™ COVID-19 Plus RealAmp Kit (OSANG Healthcare Co., Ltd., Gyeonggi-do, Korea).

In this study, all nasopharyngeal samples should have a Ct value <35 thermal cycles for inclusion in the study of ocular RNA viral analyses. Ocular samples were defined as positive when the Ct values were up to 40.

Data were presented as mean (standard deviation [SD]) or frequency (proportion) in contingency tables. Comparative analyses were conducted using Student’s t-test or Mann-Whitney U test for continuous variables. For categorical variables, the chi-squared or Fisher exact tests were used. All analyses were performed with Stata version 14 (College Station, TX, USA). All p-values <0.05 were considered statistically significant.

## RESULTS

A total of 152 patients were included in the study. The mean age (SD) was 37.0 (11.1), and the majority of patients were female (67.7%). The nasopharyngeal PCR test was positive in 86 (56.6%) patients. Table 1 shows the baseline characteristics, general symptoms, and ocular manifestations of the total study population stratified by the nasopharyngeal PCR status.

**Table 1:** Baseline characteristics and ocular signs and symptoms of the study population stratified by the Schirmer and swab/cytology status

	Schirmer test (n=152)		p-value	Swab/cytology test (n=152)		p-value
	Negative (n=138)	Positive (n=14)		Negative (n=137)	Positive (n=15)	
Age, years	35.9 (10.5)	47.0 (11.7)	0.001	36.4 (10.8)	42.2 (12.9)	0.053
Sex, (%)			0.381			0.083
Female	95 (92.2)	8 (7.8)		96 (93.2)	7 (6.8)	
Male	43 (87.7)	6 (12.3)		41 (83.7)	8 (16.3)	
Duration of symptoms, days	3.9 (1.9)	4.5 (2.2)	0.352	4.1 (1.9)	3.3 (1.6)	0.147
Ocular signs/symptoms (%)						
Redness			0.153			0.998
No	113 (92.6)	9 (7.4)		110 (90.1)	12 (9.9)	
Yes	25 (83.3)	5 (16.7)		27 (90.0)	3 (10.0)	
Tearing			0.002			0.248
No	99 (96.1)	4 (3.9)		95 (92.2)	8 (7.8)	
Yes	39 (79.6)	10 (20.4)		42 (85.7)	7 (14.3)	
Photophobia			0.495			0.998
No	110 (91.6)	10 (8.4)		108 (90.0)	12 (10.0)	
Yes	28 (87.5)	4 (12.5)		29 (90.6)	3 (9.4)	
Eye discharge			0.658			0.998
No	123 (91.1)	12 (8.9)		121 (89.6)	14 (10.4)	
Yes	15 (88.2)	2 (11.8)		16 (94.1)	1 (5.9)	
Itching			0.239			0.775
No	93 (93.0)	7 (7.0)		91 (91.0)	9 (9.0)	
Yes	45 (86.5)	7 (13.4)		46 (88.4)	6 (11.6)	
Foreign body sensation			0.999			0.999
No	117 (90.7)	12 (9.3)		116 (89.9)	13 (10.1)	
Yes	21 (91.3)	2 (8.7)		21 (91.3)	2 (8.7)	
Changes in visual acuity			0.092			0.999
No	138 (91.4)	13 (8.6)		136 (90.1)	15 (9.9)	
Yes	0 (0.0)	1 (100.0)		1 (100.0)	0 (0.0)	
Eyelid edema			0.022			0.030
No	123 (93.2)	9 (6.8)		122 (92.4)	10 (7.6)	
Yes	15 (75.0)	5 (25.0)		15 (75.0)	5 (25.0)	
Follicular conjunctivitis			0.567			0.779
No	90 (91.8)	8 (8.2)		89 (90.8)	9 (9.2)	
Yes	48 (88.9)	6 (11.1)		48 (88.9)	6 (11.1)	

* Data are presented as mean (standard deviation) or frequency (proportion).

No differences in the mean age and sex distribution were found between the nasopharyngeal PCR groups. A higher proportion of the patients had fever, cough, and loss of taste and smell with positive nasopharyngeal PCR tests. The mean times of symptom onset were 4.1 and 3.9 days for the positive and negative nasopharyngeal PCR, respectively, and no statistical difference was found between them (p=0.608). Regarding ocular signs and symptoms, a significantly higher proportion of negative nasopharyngeal PCR tests were found in those with itchy eyes.

Both ocular surface collection techniques detected viral particles in the tears of the study participants. The Schirmer test was positive in 16.3% (14/86) of the patients with positive nasopharyngeal PCR test, and the conjunctival swab/cytology test was positive in 17.4% (15/86) of the patients with positive nasopharyngeal PCR tests. No positive ocular tests were found in those with negative nasopharyngeal PCR tests. Table 2 shows the positivity of the Schirmer and conjunctival swab/cytology tests compared with the nasopharyngeal PCR test. Moreover, the sensitivity rates of the Schirmer and conjunctival swab/cytology tests were 16.3% and 17.4%, respectively. Both ocular tests presented 100% specificity. The overall agreement between the two ocular tests was 92.7%. If these tests were combined, i.e., considering the positivity of either test, the sensitivity would be 23.2%, and the specificity would remain 100%.

**Table 2:** Positivity for Schirmer and cytology PCR tests compared with the nasopharyngeal (naso) PCR test for COVID-19

	Naso PCR	
	Negative	Positive
Schirmer		
Negative	66 (100.0)	72 (83.7)
Positive	0 (0.0)	14 (16.3)
Total	66 (100.0)	86 (100.0)
Cytology		
Negative	66 (100.0)	71 (82.6)
Positive	0 (0.0)	15 (17.4)
Total	66 (100.0)	86 (100.0)

* Data are presented as frequency (proportion).

**Figure 1:**
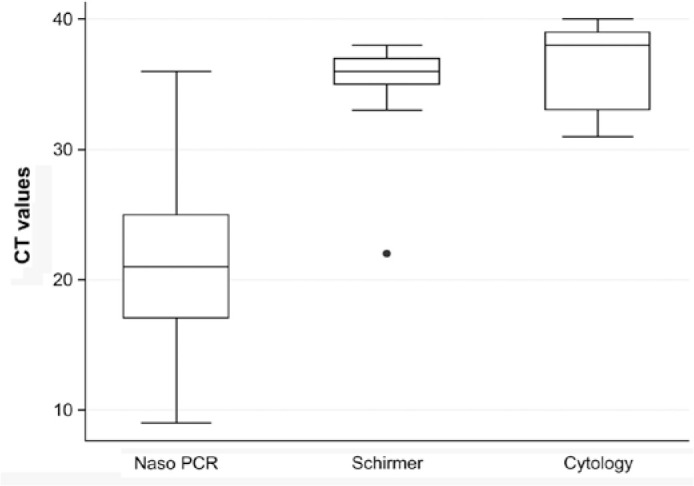
Boxplot showing the cyclic threshold (Ct) values in patients with positive reverse-transcriptase polymerase chain reaction for the nasopharyngeal, Schirmer, and conjunctival swab/cytology tests.

The Ct values of the ocular tests and nasopharyngeal PCR were also compared. No differences were found between the mean Ct value for the Schirmer test (35.6; ±1.4 cycles) and conjunctival swab/cytology test (36.4; ±3.9 cycles) (p=0.591). A statistically significant difference was found between the Schirmer (p=0.001) and conjunctival swab/cytology (p<0.001) tests when compared with the nasopharyngeal PCR test (18.2; ± 5.3 cycles) (Figure 1).

No differences were found in the positivity of systemic symptoms in both Schirmer and conjunctival swab/cytology tests. Table 1 shows the baseline characteristics and ocular signs and symptoms stratified by the ocular test status. In the stratification according to the results of the Schirmer test, those who presented with tearing (p=0.002) and eyelid edema (p=0.022) were more likely to have a positive test than those without these signs/symptoms. Patients who presented with eyelid edema had a higher proportion of positive eye conjunctival swab/cytology test results (p=0.030) than those without edema.

## DISCUSSION

This study included 152 patients with suspected COVID-19, of which 86 (56.6%) presented positive nasopharyngeal PCR test for SARS-CoV-2, with a mean period of 4.5 days from symptom onset, a period in which the probability of detecting SARS-CoV-2 infection through nasopharyngeal PCR is favorable. Nasopharyngeal PCR positivity can be found from the first day of symptom onset, peaking in the first week (5-7 days), declining until the third week, and becoming negative thereafter^(10,11)^. No differences were found in the mean symptom duration between the PCR groups (p=0.608).

The most frequent systemic clinical findings in our study were fever, cough, loss of taste, and smell. Our findings are in accordance with those already reported by Lovato et al. in a systematic review of the literature on COVID-19^(12)^.

Extensive effort has been conducted to elucidate the possibility of the ocular surface as a transmission route for SARS-CoV-2. Accordingly, many publications have demonstrated high variability of viral RNA detection on the ocular surface. Emparan et al. reported variable PCR positivity, ranging from 0% to 7.14% in both tears and conjunctival swab^(7)^. Karimi et al. found 7% positivity in conjunctival swabs of patients with confirmed diagnosis by nasopharyngeal PCR^(13)^. Arora et al. detected viral particles in the ocular surface of 24% of patients with moderate-to-severe COVID-19^(14)^. This high variability was initially attributed to disease severity; however, it was more associated with disease onset in which molecular tests proceeded^(11,15)^. Moreover, small amounts of SARS-CoV-2 RNA have also been attributed to the low sensitivity of RT-PCR^(16)^. Specifically, in relation to the ocular surface, the sample can be of a small amount and, still, be diluted by the tear^(17)^. The majority of published studies in the field used conjunctival swabs and/or Schirmer strips for sampling^(18-21)^. To overcome the heterogeneity of clinical severity in the study population, our inclusion criteria were defined to investigate the presence of SARS-COV-2 viral particles in the ocular surface of outpatients with clinical suspicion of COVID-19. Therefore, the main measurement used that allowed for the uniformity of the study population was the time of symptom onset for nasopharyngeal PCR diagnosis (4.5 days, SD, 2.2).

Conjunctival swab has been considered the gold standard for the evaluation of viral RNA^(14)^. To determine the more accurate method of preocular film viral RNA search, we conducted sampling randomly as previously described. We conducted the nasopharyngeal PCR and ocular test in a single time point of collection and used the same viral detection kit for both nasopharyngeal and ocular PCR tests. This allowed us to analyze the correlation between the Ct values of nasopharyngeal and ocular surface samples. We also analyzed the accuracy (sensitivity and specificity) of both ocular surface sampling methods based on the nasopharyngeal PCR status. To the best of our knowledge, these approaches of ocular surface sampling for SARS-CoV-2 have not been compared randomly based on the nasopharyngeal PCR status.

Both Schirmer and conjunctival swab/cytology tests detected SARS-CoV-2 particles in the ocular surface of patients with positive nasopharyngeal PCR tests (16.3% and 17.4%, respectively). No positive ocular tests were obtained among those with negative nasopharyngeal PCR tests. Thus, the sensitivity of the Schirmer test was 16.3%, and that of the conjunctival swab/cytology test was 17.4%. Both ocular tests presented 100% specificity. Importantly, both tests demonstrated an agreement of 92.7%, and their combination, considering positivity of either one, would increase the sensitivity to 23.2%, and specificity would remain at 100%. Our results are similar to those reported by Arora et al.^(14)^ They analyzed tear film samples from 75 patients categorized into three groups (group 1, conjunctival swab plus Schirmer’s test strips; group 2, conjunctival swab; and group 3, Schirmer’s test strips) and reported an overall positivity of 24% considering the method tested. They found 14.7% positivity when pooling material from the Schirmer strips and conjunctival swabs (group 1), 14.7% positivity from the conjunctival swabs (group 2), and 9.3% positivity from the Schirmer strips (group 3). We analyzed samples from 152 patients and found higher positivity in both conjunctival swabs (17.4% vs 14.7%) and Schirmer strips (16.3% vs 9.3%). We speculate that our higher sensitivity might be related to the immediate storage of our samples in 150 µL of storage and stabilization solution (DNA/RNA Shield), avoiding a dilution factor in the buffer solution that could consequently reduce the amount of viral genetic materials in a sample. However, our positivity from a single test was still higher (14.7% vs 16.3% for the Schirmer test and 14.7% vs 17.4% for the conjunctival swab test). Importantly, ocular surface/preocular film positivity and/or sensitivity and specificity for SARS-CoV-2 should be treated with caution when compared with oropharyngeal and nasopharyngeal PCR tests that are more sensitive and appropriate for diagnosis.

Another interesting finding was the Ct values in both nasopharyngeal and ocular samples. Although not usually comparable between assays, it is acceptable that the higher the viral load, the lower the CT value. Many studies have correlated Ct values from nasopharyngeal PCR with clinical severity and infectiveness^(15,22)^. We defined our Ct cutoff for positive ocular samples at 40 cycles. Ct values for ocular RT-PCR were 35.6 and 36.4 for the Schirmer test and conjunctival swab/cytology test, respectively. No statistical difference was found between the methods (p=0.591). Accordingly, this reflects similar positivity and sensitivity between both Schirmer and conjunctival swab/cytology tests previously mentioned in our study.

In our series, a nasopharyngeal PCR Ct value of 18.2 was significantly lower than that in both ocular surface tests and therefore indirectly demonstrated that the viral load was lower in the ocular surface than in the upper respiratory tract. Only the study by Arora et al. mentioned the Ct values of positive tear samples (cutoff set for 35 cycles) with no statistical difference among the methods they have tested (28.36 ± 6.15 in pooled material from Schirmer strips and conjunctival swabs, 29.00 ± 5.58 in conjunctival swabs, and 27.86 ± 6.46 in Schirmer strips). However, they could not correlate nasopharyngeal Ct values with ocular surface Ct values in their series because of the different collection times^(14)^. We are unaware of any previous study that correlated Ct values from the nasopharynx simultaneously with positive tear samples, supporting the lower viral shedding in the ocular surface. A few mechanisms could explain the lower viral load in the ocular surface: (1) the small amount of ACE2 receptor and TMPRSS2 in the ocular surface, known as required elements for SARS-CoV-2 adhesion to host cells, compared with the upper respiratory tract^(19)^; (2) a tear film innate immunity protecting the ocular surface constituted by lactoferrin, lysozyme, and lipocalin that have proven their role against other viral infections but not yet fully elucidated against SARS-CoV-2^(23-25)^; (3) the IgA present in the tear film and ocular surface that binds to the spike protein of SARS-CoV-2, decreasing viral invasion^(26)^; and (4) the blink mechanism that continuously wash microorganisms from the ocular surface^(27)^.

Regarding ocular signs and symptoms, a higher ocular surface positivity for COVID-19 would be associated with conjunctivitis and/or more inflamed eyes. However, some studies have demonstrated that SARS-CoV-2 positivity could not be related to ocular signs and symptoms^(9,14)^. In this study, we analyzed systemic and ocular signs and symptoms according to the nasopharyngeal PCR status and according to both ocular surface tests. No differences were found in the positivity of both Schirmer and conjunctival swab/cytology tests regarding the most frequent systemic clinical features (fever, cough, loss of taste, and smell symptoms). The most frequent ocular signs and symptoms based on the nasopharyngeal PCR status were follicular conjunctivitis (54/152; 35.5%), itching (52/152; 34.2%), tearing (49/152; 32.2%), photophobia (32/152; 21.05%), and redness (30/152; 19.7%). However, they were not clearly associated with ocular PCR positivity. Accordingly, Dutescu et al. and Arora et al. have reported that SARS-CoV-2 positivity would not necessarily be correlated to ocular signs and symptoms^(8,14)^. However, they included hospitalized patients with moderate-to-severe COVID-19 that could not undergo slit lamp biomicroscopy. In our series, all patients underwent slit lamp biomicroscopy evaluation by an ophthalmologist to recognize signs and symptoms associated with viral conjunctivitis in outpatients during the first week of systemic symptoms. The analysis of ocular signs and symptoms stratified by Schirmer test results demonstrated that those who presented tearing (p=0.002) and eyelid edema (p=0.022) were more likely to have a positive test than those without these signs and/or symptoms. Eyelid edema was also associated with a higher proportion of positive eye conjunctival swab/cytology test results (p=0.030) than those without edema. Importantly, although not associated with ocular PCR status, a few signs and symptoms such as follicular conjunctivitis, tearing, foreign body sensation, eyelid edema, and itching were relatively frequent in our series. Thus, all patients included in this study presented flu-like symptoms and therefore their ocular findings could be associated with other viral infections of the upper airway tract distinguished from SARS-CoV-2. Among patients with negative nasopharyngeal PCR, itching was significantly reported. Again, this could be related to other viral infections, such as adenovirus. We also speculate that the chronic use of facemasks causes itchy eyes, mimicking dry eye-related complaints, as this has already been reported by a few studies^(28-30)^.

Although this has been the largest series of different modalities for SARS-CoV-2 assessment in the ocular surface, this study was limited by the small sample size and one-time sampling design in the acute phase of COVID-19 in outpatients. By contrast, it allowed slit lamp ophthalmological examination to identify ocular signs and compare viral load between nasopharyngeal PCR and ocular PCR tests that were simultaneously collected and promoted a deeper discussion of ocular surface test accuracy to detect SAR-CoV-2. Importantly, oropharyngeal and nasopharyngeal PCR tests are clearly more sensitive and appropriate for diagnosis. Another concern is the use of anesthetic eyedrops as a dilution factor during conjunctival swab; however, sampling the inferior conjunctival fornix with a cervical brush can cause patient discomfort and that justify its use, which has been previously recommended by the institution’s ethics committee. On the contrary, insufficient tear collection could interfere with the positivity rate when using the Schirmer strip filters, but we achieved the minimum cutoff of 15 mm in all patients. Viral shedding in the ocular surface and its role in the diagnosis, infectiveness, and care of patients with COVID-19 deserve further investigation.

Our results demonstrated that both Schirmer and conjunctival swab tests were comparably capable of detecting SARS-CoV-2 RNA in the ocular surface by real-time RT-qPCR analysis. They were also comparably accurate based on the nasopharyngeal status and demonstrated indistinct sensitivity and specificity. Simultaneous specimen sampling and processing from the nasopharyngeal, Schirmer, and conjunctival swab tests demonstrated significantly lower viral load in both ocular surface approaches than that in the nasopharyngeal approach. Ocular manifestations detected by slit lamp biomicroscopy were not clearly associated with ocular real-time RT-PCR positivity.

Viral load in the ocular surface and its role in the diagnosis, infectiveness, and care of patients with COVID-19 should be further investigated. However, considerable evidence recommends the judicious use of individual protective equipment, such as goggles and face shield, by healthcare workers when interacting with patients with COVID-19.
